# Striving Towards Equity in Cardiovascular Genomics Research

**DOI:** 10.1007/s11883-025-01277-z

**Published:** 2025-02-18

**Authors:** Javier Jurado Vélez, Nekayla Anderson, Ivree Datcher, Christy Foster, Pamela Jackson, Bertha Hidalgo

**Affiliations:** 1 Marnix E Heersink School of Medicine, University of Alabama at Birmingham, Birmingham, AL USA; 2 Department of Epidemiology, School of Public Health, University of Alabama at Birmingham, Birmingham, AL USA; 3 Division of Pediatric Endocrinology and Diabetes, Department of Pediatrics, University of Alabama at Birmingham, Birmingham, AL USA; 4 Department of Environmental Health Sciences, School of Public Health, University of Alabama at Birmingham, Birmingham, AL USA

**Keywords:** Cardiovascular genomics, Health equity, Ancestry, Genetic variability, Genetics, Cardiovascular disease

## Abstract

**Purpose of Review:**

Our review emphasizes recent advancements and persisting gaps in cardiovascular genomics, particularly highlighting how emerging studies involving underrepresented populations have uncovered new genetic variants associated with cardiovascular diseases.

**Recent Findings:**

Initiatives like the *H3Africa* project, the Million Veterans Program, and the *All of Us* Research Program are working to address this gap by focusing on underrepresented groups. Additionally, emerging research is centering on the interplay between genetic factors and socio-environmental determinants of health, which disproportionately impact marginalized communities.

**Summary:**

As cardiovascular genomics research grows, increasing the inclusion of underrepresented populations is essential for gaining a more comprehensive understanding of genetic variability. This will lead to more accurate and clinically meaningful strategies for preventing and treating cardiovascular diseases across all ancestral backgrounds and diverse populations.

## Introduction

- Overview of Cardiovascular Genomics
- Health Equity in Genomics Research
- Purpose of the Review

Genomics research has its roots in clinical observations of rare diseases, such as dilated cardiomyopathy or *FNB1*, the gene that causes Marfan syndrome [1]. These early observations identified genes that followed a Mendelian pattern of genetics. Mendelian genetics refers to the inheritance patterns of traits that are determined by single genes, where traits are passed from parents to offspring in predictable ways based on dominant and recessive alleles. However, the field of cardiovascular genomics expanded significantly with the publication of the HapMap project in 2003 and advent of genome-wide association studies (GWAS), which aided in the identification of genetic variants associated with more common, complex diseases including cardiovascular diseases [2, 3]. Familial studies enabled observations of associations of family heritability, and the emergence of GWAS and whole-genome sequencing (WGS) enabled researchers to pinpoint genetic variants that connected heritability with cardiovascular diseases (CVDs) [2, 4]. Modern clinical guidelines have since recognized at least 30 genetic variants associated with various CVDs, allowing healthcare professionals (HCPs) to test patients at high risk for certain diseases and target management in diseases such as cardiomyopathies or long QT syndrome [5].

In 2022, CVD accounted for 19.8 million deaths globally, and remains the leading cause of death and a critical public health issue [6]. Age-standardized CVD mortality was highest in Eastern Europe, Central Asia, the Middle East, and most of the African continent [6]. However, the patient population for GWAS studies consists of 78–91% of patients from European ancestry, lacking an inclusive representation of underrepresented communities [7–9]. Although progress has continued, calls to action have prompted health equity to remain an ongoing goal throughout all forms of genomic research [10–13]. Data used in GWAS studies often lack adequate representation of diverse ancestral groups. This underrepresentation can introduce biases in genetic findings, ultimately leading to clinical guidelines that fail to account for the needs of broader, more diverse patient populations [2, 7, 14–16]. To address these disparities, it is essential to carefully define how we categorize individuals in genetic research. Race and ethnicity are socially constructed categories that individuals typically self-identify with; they do not reflect inherent biological differences. In contrast, ancestry in genetic research refers to an individual’s biological lineage, inferred from their DNA. Understanding this distinction is critical to ensuring that genetic studies accurately capture human diversity and do not reinforce misleading biological interpretations of race and ethnicity [17].

The inclusion of diverse populations will address remaining gaps of diverse ancestral backgrounds in genomic study populations, while those at increased risk for CVD will reap the benefits of diversity-enhanced genomic research [10, 11]. Several global initiatives have been established to recruit participants from underrepresented communities to provide more data from missing ancestral backgrounds, which will bolster genomics data [15, 18–21]. This review aims to highlight the current landscape of genetic and epigenetic studies, emphasizing how bridging the gaps in genomic data will facilitate more accurate, personalized medicine for diverse populations, ultimately enhancing prevention and treatment strategies for cardiovascular disease.

### The Current Landscape of Cardiovascular Genomics Research

- Recent Advances in Cardiovascular Genomics
- Technological Innovations

Significant advances and technological innovations in cardiovascular genomic research have improved the accuracy of genomic data. Epidemiological studies have consistently demonstrated that family history is a major risk factor for CVD [2, 5], with heritability estimates as high as 50% [22]. Well-known heritable diseases, such as long QT syndrome and dilated cardiomyopathy, have been investigated through observational studies, and genomics research has identified genetic variants associated with these diseases [23, 24]. Moreover, the pathophysiology of congenital heart diseases remains elusive, but genetic studies have presented some probable explanations [25–27]. Mutations in the genes *GATA4* and *NKX2-5*, which encode transcription factors crucial for the expression of cardiac natriuretic peptides and cardiac development, have been strongly associated with cardiac defects, including atrial septal defects (ASD), ventricular septal defects (VSD), and double outlet right ventricle (DORV), among others [25]. Deletion involving *TBX1*, an important transcription factor, can result in the disruption of pharyngeal pouch development, leading to a 22q11.2 deletion syndrome (del22q11) phenotype, commonly known as DiGeorge syndrome [28]. Epigenetic studies have also reported that maternal smoking and alcohol consumption can impact the expression of these transcription factors [26, 27]. The TAMRISK study, a Finnish population of 320 patients with hypertension and 439 control patients, found a significant association between hypertension and a single nucleotide polymorphism (SNP) **rs2779249** in the inducible nitric oxide synthase (*iNOS*) gene [29]. Notably, study participants with the rare A allele in the gene had a greater risk of hypertension as early as 35 years old [29].

Clinical applications of cardiovascular genomic research include earlier testing, diagnosis, and medical management of high-risk patients or those with known family history [15, 30]. For example, familial hypercholesterolemia (FH), a genetic mutation in low-density lipoprotein (LDL) receptors, can lead to extremely high LDL cholesterol levels among patients with the genetic mutation. FH has also been associated with an extremely high risk for CVD outcomes [15, 30]. Advances in genomic research will reveal further evidence to help decrease the risk of CVD outcomes among diverse communities.

Genomics research is also a burgeoning area in pharmacology. While early-onset use of statin medications has proven to reduce the risk of negative cardiovascular outcomes [4], specific pharmacotherapy guidelines (based on genomics research) have been developed to address the influence of genetic variations in responses to statin therapy (i.e., *SLCO1B*1 gene mutations) [31]. Pharmacogenetic research has also become more relevant as genetic variants protective against CVDs have influenced the development of new cardiometabolic drugs [32]. Individuals with an absent *APOC3* gene, which encodes apolipoprotein C3, an inhibitor of lipoprotein lipase and triglyceride hepatic uptake, have a blunted post-prandial spike in plasma triglycerides [33]. This observation has led to new drugs currently undergoing clinical development [34]. Several mRNA-targeting therapies developed are highly effective in targeting the expression of lipoprotein(a), leading to significantly reduced LDL levels [32]. Two drugs, Pelacarsen and Olpasiran, are currently undergoing Phase III clinical trials, with results anticipated within the next two years [35, 36].

Genomics research has also created significant advances in the creation of Polygenic Risk Scores (PRS), a tool developed to estimate an individual’s risk for certain diseases based on their genotype profile and known GWAS data [37, 38]. Initial studies examining PRS related to coronary heart disease (CHD) showed poor reliability [39]. However, the expansion of genomics data has led to multiple studies creating more precise PRS estimations to accurately predict a patient’s risk for CHD [40, 41]. This is also significant given that patients with high-risk PRS experience a greater benefit from statin use compared to those with low-risk PRS [4].

### Inequities in Cardiovascular Genomics

- Epidemiology of Cardiovascular Diseases
- Social Determinants of Health

As noted previously, although significant progress has been made, crucial limitations in our current cardiovascular genomics data remain. Many landmark studies for the PRS of CHD used European or Asian ancestry, lacking robust samples from populations of African and Indigenous ancestry for PRS development [2, 4, 15]. Furthermore, limited sample diversity decreases the generalizability and application of these results to genetic testing in other populations. Lack of generalizability due to ancestral background has been demonstrated in the past [7, 10, 11, 16, 18, 19]. One of the earliest loci found in cardiovascular genomics was the 9p21 locus, which was strongly associated with coronary artery disease (CAD). However, research has shown that the 9p21 locus has varying effects across different ancestries [39, 42]. While it is true that the association was initially strongest in European populations, subsequent studies have found that the locus also has associations with CAD in non-European populations, including some African, South Asian, and East Asian populations, though often with differing effect sizes or degrees of statistical significance [43, 44]. However, these associations tend to be weaker in African populations, likely due to differences in allele frequency or genetic architecture. Though increasing in number, few studies have identified loci associated with CAD among ancestries underrepresented in genomics research [42, 45, 46]. A meta-analysis of multi-ethnic biobanks was performed to assess the performance of PRS for CAD across different ethnicities. Each ancestral group had a statistically significant association between PRS and CAD and statistically significant variability in effect size across ancestries. European and East Asian ancestries performed best, with an odds ratio of 1.60 (95% CI, 1.44–1.78) and 1.66 (95% CI, 1.47–1.86), respectively. On the other hand, the African ancestry PRS performed the worst, with an odds ratio of 1.25 (95% CI, 1.12–1.40), highlighting the need for more diverse GWAS and continuing efforts to improve PRS performance [46].

Increasing diversity among participants in genomics research will enhance the representation of SNPs, including rare variants, which are essential for identifying associations with cardiovascular disease [2]. Ancestries underrepresented in genomics research, such as Indigenous American and Hispanic or Latin American populations, often have heterogeneous genetic backgrounds [42, 47]. It is also important to recognize that race and ethnicity are social constructs rather than biological categories. Therefore, while genetics plays a role in disease risk, lived experiences, and social inequalities—often shaped by racial and ethnic categorizations—can influence epigenetic modifications such as DNA methylation, further impacting CVD risk [48–50]. It is important to consider how lived experiences and social inequalities among diverse populations can impact their DNA and, in turn, further increase their genetic variability.

Racial and ethnic minority groups are more likely than White individuals to live in segregated neighborhoods, where they are exposed to disadvantages and stressors that contribute to health disparities [51]. Social epigenetics has revealed that epigenetic changes related to these social and lived experiences can impact health inequalities. Shields et al. identified increased DNA methylation at the *NR3C1* gene among Black women who experienced abuse during their childhood, with increasing methylation correlated to increasing severity of abuse [52]. In Black men, advantageous ties with peers, parents, and partners were inversely associated with DNA methylation at *OXTR*, a gene encoding the oxytocin receptor, leading to less substance abuse [53, 54]. Even experiences of discrimination are associated with increased methylation in genes related to stress (i.e., *NR3C1*, *BDNF*, *FKBP5*) and inflammation (*LRRN3*) among Black and Latinx populations [51].

### Overcoming Barriers Towards Greater Participation among Underrepresented Populations

- Inclusion of Diverse Populations
- Barriers to Inclusion
- Efforts to Improve Inclusion

Barriers to increasing diversity in genetic research participants are well recorded. These barriers are multifactorial, including lack of research among these cohorts and small sample size availability when recruiting from underrepresented populations, which can lead to difficulty in securing funding for research with greater diversity. Unfortunately, these challenges have also affected the collection of genetic samples as well [7]. A significant barrier is the lack of trust, rooted in a history of exploitation and betrayal by researchers toward minority communities [7, 14]. Historical transgressions such as the brutal Tuskegee Study of Untreated Syphilis and the collection and mass production of HeLa cells without consent or knowledge from Henrietta Lacks’ family have instilled a fear of harm and mistrust of the scientific community among Black populations [55]. This distrust is especially concerning in genetic research, as biological samples hold profound cultural significance for many communities [7]. Additionally, some belief systems view genetic material as an extension of the self and a connection to ancestors, further complicating participation in genomic studies [7].

Individuals from underrepresented populations are also less likely to access health care, which is a primary source for researchers to recruit patients into their study populations [7, 56]. These communities are more likely to face greater obstacles when trying to access health care, including access to transportation, time constraints, or financial difficulties [7]. Additionally, insufficient efforts by researchers to engage with minority communities outside of healthcare settings can limit awareness and interest in research participation. As a result, individuals from these populations may be less informed about available studies or opportunities for involvement [36]. Increasing the diversity of research staff and persons with diverse lived experiences can introduce valuable cultural perspectives and improve targeted recruitment strategies, ultimately enhancing the diversity of study populations [7, 56, 57].

Fortunately, several global initiatives have been launched to increase the diversity of genomics data. New guidelines and ethical codes for genomics research, such as the *International Ethical Guidelines for Health-Related Research Involving Humans*, have been established to promote greater trust in genetic research [58]. The Global Alliance for Genomics and Health constructed ethical policies and a framework for sharing genetic and health data [59]. Additionally, various organizations are advocating for greater cultural humility in genomics research through shared policies [14, 60–62]. In response to long-standing calls for more diversity, large biobanks have begun addressing the lack of representation in their data, and new projects have been initiated to build data banks specifically designed to include underrepresented populations [7, 63, 64]. For example, the *Silent Genomes Project* in Canada and the *Aotearoa Variome* in New Zealand focus on creating databases for Indigenous populations [7], while the Women’s Genome Health Study has recruited more than 28,000 women in the US to identify genetic variants in major health outcomes for women [18]. Other efforts like the All of Us Research Program and the Million Veterans Program, aim to recruit patients from different genders and ethnic backgrounds throughout the US [65, 66]. Notably, the All of Us Research Program has already improved inclusion of underrepresented communities, especially with Non-Hispanic Black individuals [67]. The publication of statements and frameworks from larger organizations such as the WHO and American College of Medical Genetics and Genomics (ACMG) have also contributed valuable insights to promote greater equity initiatives in genomics research [17, 68–70].

### Recent Findings in Cardiovascular Genomics with Implications for Health Equity

- Genetic Variants and Health Disparities
- Gene-Environment Interactions

The field of cardiovascular genomics is still relatively new, making the current emphasis on increasing genetic diversity early in its development especially promising. This focus is crucial if cardiovascular genomics is to effectively guide personalized medical management. For instance, Landry and Rehm found that genetic testing for cardiomyopathy yields significantly lower detection rates and higher rates of inconclusive results in underrepresented populations [71]. This discrepancy likely arises due to the underrepresentation of these populations in cohorts and consortia with existing genetic data. Additionally, the genetic architecture of cardiomyopathy may differ across populations, with certain pathogenic variants being more common in some ancestry groups than others. Smaller sample sizes for underrepresented populations in genomic studies further limit variant classification, reducing the statistical power needed to establish disease associations. Expanding genetic studies to include diverse populations and developing ancestry-specific reference databases will be essential for improving diagnostic accuracy and ensuring equitable benefits from cardiovascular genomics.

Studies of Indigenous populations have revealed extensive genetic diversity, even within distinct communities, due to their rich ethnic backgrounds [6, 72]. For example, Mestizo/Amerindian populations have genetic variants on *MTHFR, GSTP1*, and *GPIIIa* genes that increase their risk of hypertension while individuals from the Oji-Cree tribe share variants in the *CPB2* gene which are associated with both blood pressure and thrombosis [72]. Several studies have reported increased rates of various congenital heart defects across Aboriginal populations, such as septal defects in Inuit and Nunavik populations or total anomalous pulmonary venous among the First Nations of Northern Ontario and Eastern Manitoba. However, there remains a significant gap in the literature linking these findings to genetic studies [72].

In contrast, some studies have identified common genetic variants across Indigenous communities. For example, a collaborative study gathered genetic data among 3324 Inuit adults living in the United States (US), Canada, and Greenland with extreme lipid profiles. They found that a common mutation in LDL receptors, the p.G116S, was associated with a 3.02-fold increased risk of hypercholesterolemia [69]. Studies using data from the Strong Heart Study, which collected genetic data from participants across 13 Indigenous tribes in the US, identified genetic variants in the *MBL* gene, which involves the creation of mannose-binding lectin (MBL), a protein involved in the innate immunity. These variants result in lower levels of MBL that correlate to a higher risk of CAD [73, 74].

The Dallas Heart Study (DHS) has also been instrumental in cardiovascular genomics, providing a rich dataset of 6,101 participants, 54% of whom are Black [75]. Landmark studies using the DHS database have examined conditions such as hypercholesterolemia, liver disease, and cardiovascular genomics, among others [76–80]. For example, Hackler et al. compared cardiac biomarkers by race and sex and found that Black individuals had higher levels of certain biomarkers related to CVD and more frequent cardiovascular events compared to White individuals [78]. One key biomarker, lipoprotein(a) [Lp(a)], was strongly associated with both race and CVD, independent of sex. Black men also had higher levels of high-sensitivity cardiac troponin T (hs-cTnT), a biomarker for subclinical myocardial injury, which increases the risk of death and myocardial infarction [81]. Identifying these biomarkers, particularly those prevalent in underrepresented populations, underscores the need to discover genetic variants that contribute to elevated risk in high-risk patients [82, 83]. Data from DHS was also crucial in the discovery of 2 nonsense mutations (Y142X, C679X) in the *PCSK9* gene among African ancestry that causes a 40% reduction in LDL levels [77]. This discovery led to the development of PCSK9 inhibitors, now widely used to manage hypercholesterolemia across various ethnic groups [84, 85].

Another significant finding is the identification of the V122I variant in transthyretin (TTR) amyloidosis, first discovered in individuals of African ancestry. This variant increases the risk of transthyretin amyloid cardiomyopathy (ATTR-CM) and related heart failure. Damrauer et al. used data from the BioMe biobank, linked to electronic health records, to explore the association between the V122I variant and heart failure risk. Their findings showed that individuals of African and Hispanic/Latino ancestry with the V122I variant had an increased risk of heart failure and cardiomyopathy. Moreover, the study revealed that these patients were often underdiagnosed or faced delays in receiving an ATTR-CM diagnosis, likely due to a lack of awareness about the variant among healthcare providers and testing delays in laboratories [19].

A meta-analysis by Armstrong et al. examined 10,700 Black participants from the Genetics of Hypertension Associated Treatments (GenHAT) and REasons for Geographic and Racial Differences in Stroke (REGARDS) studies [86]. They identified 10 significant genetic variants associated with stroke risk, most of which were previously found in European and Asian populations. However, one variant near the *TMTC1* gene had not been identified before. The study also found that PRS had lower predictive accuracy in Black populations compared to models that used established stroke risk factors.

As mentioned previously, DNA methylation studies have also become an integral part of cardiovascular genomics research. For example, research on DNA methylation and metabolic syndrome (MetS) in individuals of European and Hispanic/Latino ancestry revealed differentially methylated CpG sites associated with increased obesity, triglyceride levels, and MetS risk [87]. Future studies using the Viva la Familia (VIVA) database will explore associations between DNA methylation and obesity-related traits in Hispanic populations [87].

Cooperation across multiple consortia by integrating data from several population-based cohorts has enabled larger sample sizes and improved the identification of true gene-environment interactions related to CVD risk [54]. One such interaction involves *ADAMTS7*, a gene associated with an increased risk of atherosclerosis. Individuals carrying protective SNPs, **rs7178051 and rs3825807**, exhibit reduced *ADAMTS7* expression, which is linked to lower atherosclerosis risk [88–90]. However, studies have shown that in smokers, expression of these protective variants is significantly reduced compared to non-smokers, thereby increasing the risk of coronary artery disease (CAD). Air pollution, calculated as living distance to the nearest major road, has been associated with 2 loci (BMP8A and BMP2) correlated to PAD and one possible locus (PIGR-FCAMR) associated with CAD [91, 92]. On the contrary, improved diet and physical activity reduced LDL-raising allelic variants and enhanced HDL-raising allelic variants [93, 94]. The inclusion of broad ancestral backgrounds and the recognition that our lived environment impacts our genetic makeup will provide diverse variability to the known genomic data and substantial new information needed to discover unique genetic variants. [7, 47].

### Personal Observations and Perspectives

- Personal Insights
- Future Directions

Achieving health equity in cardiovascular genomics research remains a formidable challenge. While genomics has significant potential to uncover disease risk factors and enable personalized treatments, the persistent underrepresentation of diverse ancestries in genomic studies limits the generalizability and applicability of research findings. This underrepresentation not only exacerbates existing health disparities but also hinders the advancement of precision medicine for populations disproportionately affected by cardiovascular diseases [95].

Research by Hidalgo, Foster, and colleagues has demonstrated the effectiveness of engaging participants by first establishing trust in both digital and clinical environments. For example, recruitment during routine clinical visits has been shown to enhance participation from underrepresented groups in epigenetic studies (unpublished). In one study, 250 predominantly Black and Latina women were successfully recruited into a heart health cohort through intentional engagement strategies, including targeted messaging on Instagram [96]. These models helped address common barriers to participation, such as mistrust of research, privacy concerns, and logistical challenges like transportation and time constraints. By fostering direct communication with participants and maintaining engagement before clinical visits, recruitment challenges were significantly mitigated. Similar approaches, especially when culturally tailored, may be highly effective in cardiovascular genomics research, improving inclusivity and participation.

The National Institute of Heart, Lung, And Blood and American Heart Association have emphasized the importance of ensuring the inclusion of Indigenous peoples and marginalized groups in genetic research [95]. To expand participation, strategies that have shown promise include diversifying research teams to enhance cultural competence, leveraging community infrastructure and local leadership, and providing accessible educational opportunities about genomics [97]. These strategies, when applied consistently, have the potential to bridge the gap in participation and ensure that the benefits of genomic research extend to all populations. Looking forward, it is essential for future studies to continue developing and refining inclusive recruitment strategies. By prioritizing the equitable inclusion of diverse populations, the field of cardiovascular genomics can realize its potential in delivering precise, personalized medical interventions that address the health needs of all communities.

## Conclusion

- Summary of Key Points
- Call to Action

The future of cardiovascular genomics lies in the equitable inclusion of diverse populations. Initiatives like the All of Us Research Program and Million Veterans Program are beginning to address the gaps in genomic data, but much work remains. Expanding participation among underrepresented groups is essential for uncovering genetic variants that influence disease risk and response to treatments. Ultimately, achieving health equity in genomics will lead to more effective, personalized medical strategies and help reduce the burden of cardiovascular disease in all populations.

## Key References

- Khoury MJ, Bowen S, Dotson WD, Drzymalla E, Green RF, Goldstein R, et al. Health equity in the implementation of genomics and precision medicine: A public health imperative. Genet Med. 2022:24(8):1630–1639. 10.1016/j.gim.2022.04.009.
  - This call to action proposes the use of public health as the driver towards actionable initiatives to educate and invite populations that have been poorly represented in cardiovascular genomics research.
- Hartiala JA, Hilser JR, Biswas S, Lusis AJ, Allayee H. Gene-Environment Interactions for Cardiovascular Disease. Curr Atheroscler Rep. 2021:23(12):75. 10.1007/s11883-021-00974-9.
  - Developed from the cooperation of large consortia and newer computational technology, this review emphasizes several gene-environment interactions with CVD outcomes, establishing the foundation of a new focus in cardiovascular genomics.
- Bosco G, Mszar R, Piro S, Sabouret P, Gallo A. Cardiovascular Risk Estimation and Stratification Among Individuals with Hypercholesterolemia. Curr Atheroscler Rep. 2024:(9):537–548. 10.1007/s11883-024-01225-3.
  - This review offers an overview of the current genomic research focused on hypercholesterolemia and its use in the development of Polygenic Risk Score.
- All of Us Research Program Genomics Investigators. Genomic data in the All of Us Research Program. Nature. 2024 Mar;627(8003):340–346. 10.1038/s41586-023-06957-x.
  - This study highlights initial genomic data from the All of Us Research Program, which included 77% of the study population from historically underrepresented communities and 46% from underrepresented racial/ethnic populations. The authors validated the data by undertaking several data harmonization and quality control analyses of well-established genetic associations with 118 diseases. Their initial analyses offers hope for future projects to rely on the developing database to provide significant findings.
- Abou-Karam R, Cheng F, Gady S, Fahed AC. The Role of Genetics in Advancing Cardiometabolic Drug Development. Curr Atheroscler Rep. 2024:26(5):153–162. 10.1007/s11883-024-01195-6.
  - This review highlights how genomics has impacted four key aspects of drug development: identifying drug candidates, anticipating drug target failures, silencing and editing genes, and enriching clinical trials. Notably, the authors highlight the creation of mRNA-targeting therapies to reduce LDL levels, including Pelacarsen and Olpasiran.
- Clarke SL, Assimes TL, Tcheandjieu C. The Propagation of Racial Disparities in Cardiovascular Genomics Research. Circ Genom Precis Med. 2021:14(5):e003178. 10.1161/CIRCGEN.121.003178.
  - Several meta-analyses conducted with multi-ethnic cohorts revealed a significant disparity that exists in the use of Polygenic Risk Scores between individuals from European ancestries and African ancestries.
- Armstrong ND, Srinivasasainagendra V, Patki A, Tanner RM, Hidalgo BA, Tiwari HK, et al. Genetic Contributors of Incident Stroke in 10,700 African Americans With Hypertension: A Meta-Analysis From the Genetics of Hypertension Associated Treatments and Reasons for Geographic and Racial Differences in Stroke Studies. Front Genet. 2021:12:781451. 10.3389/fgene.2021.781451.
  - This study comprised of 10,700 African American individuals with hypertension from the GenHAT and REGARDS trials conducted a meta-analysis to uncover several biologically plausible genetic determinants for incident stroke. Although most variants were similar to previously-identified ones with European and Asian ancestries, the authors highlighted 1 newly-discovered variant near TMC1.
- Lee SS, Appelbaum PS, Chung WK. Challenges and potential solutions to health disparities in genomic medicine. Cell. 2022:185(12):2007–2010. 10.1016/j.cell.2022.05.010.
  - This paper shares the importance of greater diversity within genomic databases and highlights several tested initiatives that have led to greater participation and inclusion of underrepresented participants.

## Data Availability

No datasets were generated or analysed during the current study.
